# Small and sick newborn care during the COVID-19 pandemic: global survey and thematic analysis of healthcare providers’ voices and experiences

**DOI:** 10.1136/bmjgh-2020-004347

**Published:** 2021-03-14

**Authors:** Suman P N Rao, Nicole Minckas, Melissa M Medvedev, David Gathara, Prashantha Y N, Abiy Seifu Estifanos, Alfrida Camelia Silitonga, Arun Singh Jadaun, Ebunoluwa A Adejuyigbe, Helen Brotherton, Sugandha Arya, Rani Gera, Chinyere V Ezeaka, Abdou Gai, Abebe Gebremariam Gobezayehu, Queen Dube, Aarti Kumar, Helga Naburi, Msandeni Chiume, Victor Tumukunde, Araya Abrha Medhanyie, Gyikua Plange-Rhule, Josephine Shabini, Eric O Ohuma, Henok Tadele, Fitsum W/Gebriel, Amanuel Hadgu, Lamesgin Alamineh, Rajesh Mehta, Elizabeth Molyneux, Joy E Lawn, on behalf of the COVID-19 Small and Sick Newborn Care Collaborative Group, Helen Brotherton

**Affiliations:** 1 Department of Neonatology, St. John's Medical College Hospital, Bangalore, India; 2 Institute for Global Health, University College London, London, UK; 3 Department of Pediatrics, University of California San Francisco, San Francisco, California, USA; 4 Maternal, Adolescent, Reproductive and Child Health (MARCH) Centre, London School of Hygiene & Tropical Medicine, London, UK; 5 Kenya Medical Research Institute-, Wellcome Trust Research Program, Nairobi, Kenya; 6 Department of Reproductive, Family and Population Health, School of Public Health, Addis Ababa University, Addis Ababa, Ethiopia; 7 Department of Reproductive, Maternal, Newborn, Child and Adolescent Health, World Health Organization, Country Office Indonesia, Jakarta, Indonesia; 8 Centre for Health Research and Development, Society for Applied Studies, New Delhi, India; 9 Department of Pediatrics and Child Health, Obafemi Awolowo University, Ile-Ife, Nigeria; 10 Medical Research Council Unit The Gambia, at London School of Hygiene & Tropical Medicine, Fajara, The Gambia; 11 Department of Pediatrics, Vardhman Mahavir Medical College and Safdarjung Hospital, New Delhi, India; 12 Department of Pediatrics, University of Lagos and Lagos State University Teaching Hospital, Lagos, Nigeria; 13 Emory University Ethiopia, Addis Ababa, Ethiopia; 14 Department of Pediatrics and Child Health, University of Malawi College of Medicine and Queen Elizabeth Central Hospital, Blantyre, Malawi; 15 Community Empowerment Lab, Lucknow, India; 16 Department of Pediatrics and Child Health, Muhimbili University of Health and Allied Sciences, Dar es Salaam, Tanzania; 17 Department of Pediatrics, Kamuzu Central Hospital, Lilongwe, Malawi; 18 Medical Research Council/Uganda Virus Research Institute and London School of Hygiene & Tropical Medicine Uganda Research Unit, Entebbe, Uganda; 19 School of Public Health, Mekelle University College of Health Sciences, Mekelle, Ethiopia; 20 School of Medicine and Dentistry, Kwame Nkrumah University of Science and Technology, Kumasi, Ghana; 21 Bagamoyo Research and Training Centre, Ifakara Health Institute, Bagamayo, Tanzania; 22 Department of Pediatrics and Child Health, Addis Ababa University, Addis Ababa, Ethiopia; 23 Hawassa University, Hawassa, Ethiopia; 24 Regional Office, South East Asia, World Health Organisation, New Delhi, India

**Keywords:** paediatrics, public health, SARS, cross-sectional survey

## Abstract

**Introduction:**

The COVID-19 pandemic is disrupting health systems globally. Maternity care disruptions have been surveyed, but not those related to vulnerable small newborns. We aimed to survey reported disruptions to small and sick newborn care worldwide and undertake thematic analysis of healthcare providers’ experiences and proposed mitigation strategies.

**Methods:**

Using a widely disseminated online survey in three languages, we reached out to neonatal healthcare providers. We collected data on COVID-19 preparedness, effects on health personnel and on newborn care services, including kangaroo mother care (KMC), as well as disruptors and solutions.

**Results:**

We analysed 1120 responses from 62 countries, mainly low and middle-income countries (LMICs). Preparedness for COVID-19 was suboptimal in terms of guidelines and availability of personal protective equipment. One-third reported routine testing of all pregnant women, but 13% had no testing capacity at all. More than 85% of health personnel feared for their own health and 89% had increased stress. Newborn care practices were disrupted both due to reduced care-seeking and a compromised workforce. More than half reported that evidence-based interventions such as KMC were discontinued or discouraged. Separation of the mother–baby dyad was reported for both COVID-positive mothers (50%) and those with unknown status (16%). Follow-up care was disrupted primarily due to families’ fear of visiting hospitals (~73%).

**Conclusion:**

Newborn care providers are stressed and there is lack clarity and guidelines regarding care of small newborns during the pandemic. There is an urgent need to protect life-saving interventions, such as KMC, threatened by the pandemic, and to be ready to recover and build back better.

**Key questions**

**What is already known?**

- The COVID-19 pandemic has disrupted health systems worldwide; a recent global survey of 714 frontline maternal care providers reported effects on pregnancy, intrapartum and postpartum services; inadequate preparedness; and increased levels of stress among health personnel.
- Pandemic-associated disruptions are increasing neonatal mortality, yet small and sick newborn care is relatively new in global health and has not yet been included in global assessments of health service disruptions.
- Keeping mothers and newborns together is a core aspect of respectful care and is particularly under threat during the pandemic, including for vulnerable newborns requiring kangaroo mother care (KMC).

**Key questions**

**What are the new findings?**

- Our survey of 1120 respondents show that COVID-19 preparedness, particularly testing of pregnant women, availability of personal protective equipment (PPE), and guidelines for small and sick newborn care, are suboptimal in all regions, with most health professionals reporting higher stress levels and 85% fearing for their own health.
- Reductions in hospital births and neonatal admissions were reported in all regions in addition to compromised newborn care due to women fearing to come to hospital, reallocation of personnel and/or equipment from newborn units, and early discharge which was reported as the norm.
- Two-thirds of workers stated they would not allow mothers whose SARS-CoV-2 status is positive or unknown to practice KMC, and >20% of workers would not allow KMC even among mothers testing negative.

**What do the new findings imply?**

- Respondents reported mitigating strategies at all levels of care such as provision of adequate PPE for all health personnel; clearer guidance, particularly on non-separation of mothers and their newborns; and higher-profile messaging on benefits and safety of early KMC during the pandemic.
- Policymakers can and must do better to protect neonatal health services, support personnel and particularly ensure evidence-based practices for all mothers and newborns, including those who are SARS-CoV-2-positive.
- Our findings provide sobering insights into disrupted care for small and sick newborns across the globe; more implementation research is urgently needed, with evaluation of mitigation approaches and sharing what works to protect vulnerable newborns.

## Introduction

Each year, 2.5 million newborns die within 28 days of birth,^1^ more than 80% of whom are low birth weight (LBW, <2500 g) and particularly vulnerable.^2^ Since the adoption of the Every Newborn Action Plan by all United Nations member states in 2014^3^ with the first ever global neonatal mortality target in the Sustainable Development Goals (SDG),^4^ momentum has increased for ending preventable newborn mortality, although less emphasis has yet been placed on stillbirths.^5^ Over 78 high-burden countries have set national mortality targets to reach 12 or fewer neonatal deaths per 1000 live births by 2030.^6^ However, major inequities still lie between and within countries. Some countries are predicted to reach the SDG target over a century too late.^2^

Newborn care is a well-recognised marker of high-quality care since it is exquisitely time-sensitive, and delays of minutes can lead to death. Keeping mothers and newborns together is a core aspect of evidence-based, respectful care, including for neonates who are born preterm (<37 completed weeks of gestation) or with LBW, or both.^2 7 8^ Facility-based care of small and sick newborns has been highlighted as having potential for high-impact (>757 000 lives per year) yet low coverage and suboptimal quality of care exist due to limited investment in most low and middle-income countries (LMIC).^9^

The COVID-19 pandemic and associated nationwide restrictions risk reversing fragile gains for maternal and newborn health in the highest burdened settings.^10^ Newborns are among the most vulnerable to the indirect effects of the COVID-19 pandemic on healthcare provision. Roberton and colleagues estimated that coverage disruptions of 9.8% to 51.9% over 6 months could result in 253 500 to 1 157 000 additional under-5 child deaths in LMICs, and these estimates did not include small and sick newborn care.^11^ An observational study in Nepal reported that institutional births were halved, while labour ward neonatal mortality increased threefold during the first 2 months of the COVID-19 lockdown.^12^ A comparative analysis of lives saved by kangaroo mother care (KMC) versus mortality risk due to COVID-19 among neonates weighing ≤2000 g showed that the benefit outweighs the risk by 65-fold to 630-fold.^13^

Maternity services were affected during initial stages of the COVID-19 pandemic, and an online survey with over 700 responses from maternity workers reported reductions in antenatal and postnatal care, and a shift in birth location from hospital to home.^14^ Quality of care was also reported to be affected, most notably evidence-based, respectful care practices such as birth companions, family visitation, keeping newborns and mothers together and breastfeeding.^14^ Importantly, health personnel reported higher workload due to staff shortages and longer shifts, and increased levels of stress. This survey provided an extremely valuable picture of the challenges faced by maternity health providers during the COVID-19 pandemic. However, as noted by the authors, only 10 respondents worked in neonatal care.^14^

Small and sick newborns are among our most vulnerable citizens and have not yet been included in global assessments of disruptions during the pandemic. We conducted a global online survey to provide insights on disruptions to coverage and quality of small and sick newborn care, and to identify possible solutions to protect vulnerable newborns during the COVID-19 and similar future pandemics.

## Methods

### Study design, population and sampling

This cross-sectional study used a methodology similar to that of the previous study on maternal health impacts of COVID-19.^14^ We targeted all relevant cadres working in newborn healthcare provision, including health providers (nurses, midwives, doctors and community health workers), public health professionals and policymakers. We particularly focused on LMICs. The survey was distributed using professional member organisations and personal networks, and was widely disseminated through social media (eg, Twitter, WhatsApp, Facebook and Telegram) and at events (eg, webinars). Respondents were encouraged to share the survey with colleagues for snowball sampling.

### Questionnaire

The questionnaire was developed by a multidisciplinary team that included neonatologists, paediatricians, nurses, epidemiologists and public health professionals from diverse settings. Where relevant, we adapted from the maternal health survey.^14^ We collected data on respondents’ characteristics, including area of work, healthcare preparedness and responses to COVID-19, and the effect of the pandemic on health professionals and newborn care. We also requested respondents to list major disruptions that occurred in their work settings and solutions that should be developed to overcome these disruptions. Embedded logic skips in the survey guaranteed that only relevant questions were asked. The questionnaire was developed in English and piloted by professionals from various settings to assess face validity and clarity of wording and answer options. The final version was translated into Spanish and Bahasa (Indonesia). The English questionnaire can be found in online supplemental appendix 1. Information was presented on the landing website and respondents were requested to indicate consent by checking a box before participating.

10.1136/bmjgh-2020-004347.supp1 Supplementary data

### Data processing, missing data and analysis

We received 1389 responses between 13 July and 13 October. During data cleaning, we removed refusals to participate (n=47), responses with country missing (n=216) and those with missing answers on >90% of questions (n=6). Analysis involved descriptive statistics (frequencies and percentages) using STATA/SE V.14. Countries were aggregated by region (online supplemental appendix 2).

10.1136/bmjgh-2020-004347.supp2 Supplementary data

We conducted a qualitative analysis of free-text responses on disruptors and solutions to neonatal care. Disruptions and solutions were coded separately using NVivo V.12. Thematic analysis of free text was used to identify common themes among disruptions reported by respondents. For analyses of solutions, we conducted a framework analysis applying an adapted version of the socioecological model previously used for KMC qualitative research (Brotherton, in press) (online supplemental appendix 3). Results are presented according to the five levels of this conceptual framework: family/ caregivers, facility/ ward, health systems, community and policy. For each level, we identified disruptions and enablers/solutions to small and sick newborn care during the COVID-19 pandemic. We triangulated qualitative and quantitative data and present combined results to enrich interpretation.

10.1136/bmjgh-2020-004347.supp3 Supplementary data

## Results

### Respondents’ characteristics

**Figure 1:**
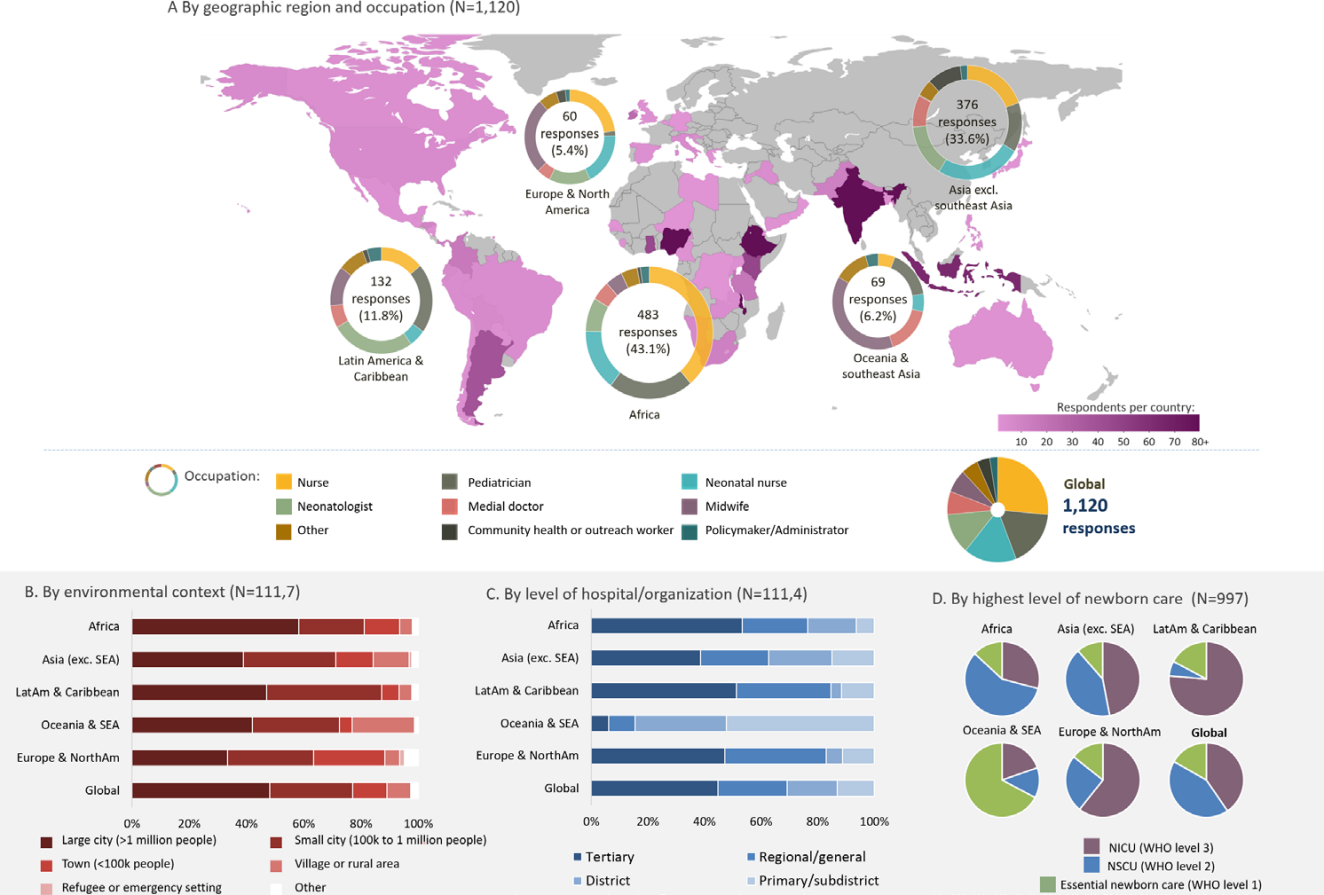
Distribution of survey respondents by country, region, occupation and hospital context. NICU, neonatal intensive care unit; NSCU, neonatal special care unit; SEA, Southeast Asia.

A total of 1120 participants (after exclusions noted above) responded to the survey, spanning 62 countries. Africa and Asia had the largest numbers: 483 (43.1%) and 376 (33.6%), respectively. Overall, the majority of the respondents were healthcare professionals: nurses (43.4%, out of which 17.0% were neonatal nurses and 26.4% not specified) and paediatricians (17.7%); policymakers and administrators constituted only 2.5% of the sample. Most respondents (>40%) across all the regions worked in tertiary level hospitals/organisations with the exception of Oceania and Southeast Asia (SEA), where 82.1% worked in primary-level or district-level facilities. Of the 997 respondents who provided direct newborn care, the majority from Africa worked in neonatal special care units (WHO level 2), while those from Asia, Latin America and the Caribbean, Europe and North America mainly provided care in neonatal intensive care units (WHO level 3).^9^ The characteristics and geographical distribution of respondents are provided in figure 1.

### COVID-19 preparedness for neonatal care

COVID-19 testing was not routinely available for pregnant women and great variations across regions were observed (table 1). While the WHO suggests that testing protocols for a pregnant woman depends on where she lives, it recommends that symptomatic pregnant women should be prioritised. Lack of testing was flagged as a major challenge by many of the respondents. Around one-third of the respondents providing facility-based care reported that SARS-CoV-2 testing was routinely available for pregnant women admitted for delivery (36.2%) and those with symptoms or contact history (30.7%). Testing of pregnant women admitted for delivery was reported as unavailable by 21.9% of respondents from Africa. Even when testing was available, the lag between testing and results impacted clinical management decisions. A neonatal nurse from India wrote, ‘*When babies are admitted in SNCU [special newborn care unit], we don't treat them until [we receive] their COVID-19 test*’. Similar experiences were reflected by other respondents who explained that practices such as KMC are postponed until maternal COVID-19 status is known. Although testing availability fluctuated across regions, 80.6% of respondents reported that facilities had sign-posted areas for COVID-19 screening and isolation.

**Table 1:** COVID-19 preparedness among neonatal providers and its impact on healthcare personnel and health systems, by region

	Africa N=334 n (%)	LatAm and Caribbean N=86 n (%)	Asia (excl. SEA) N=300 n (%)	Oceania and SEA N=66 n (%)	North America and Europe N=21 n (%)	Total* N=807 n(%)
Test for SARS-CoV-2 available at admission for delivery						
Routinely for all women	30 (9.0)	25 (30.1)	180 (62.3)	43 (67.2)	8 (38.1)	286 (36.2)
Only for women with risk factors	81 (24.3)	14 (16.9)	36 (12.5)	8 (12.5)	6 (28.6)	145 (18.4)
Only for women with symptoms or contact history	147 (44.1)	37 (44.6)	53 (18.3)	0 (0.0)	5 (23.8)	242 (30.6)
Only for elective caesarean section	2 (0.6)	0 (0.0)	5 (1.7)	3 (4.6)	0 (0.0)	10 (1.3)
Never available	73 (21.9)	7 (8.4)	15 (5.2)	10 (15.4)	2 (9.5)	107 (13.5)
Sign-posted area for SARS-CoV-2 screening available in facility	254 (76.3)	80 (94.1)	217 (82.8)	48 (73.9)	18 (85.7)	617 (80.6)
Isolation areas for suspected and confirmed SARS-CoV-2 cases available in facility	296 (88.6)	83 (97.7)	199 (75.7)	36 (56.1)	17 (89.6)	631 (82.)
Sufficient PPE items†						
Gloves	162 (49.7)	66 (79.5)	212 (75.3)	14 (21.5)	17 (85.0)	471 (59.9)
N95 masks	37 (11.7)	44 (52.4)	114 (39.7)	8 (13.3)	10 (50.0)	213 (27.6)
Eye protection	42 (13.1)	46 (55.4)	99 (35.0)	11 (17.7)	13 (65.0)	210 (27.3)
Aprons or gowns	84 (26.3)	57 (69.7)	142 (48.5)	10 (16.4)	14 (70.0)	308 (39.9)
Sanitisers	156 (48.2)	65 (77.4)	230 (79.0)	14 (21.9)	16 (84.2)	481 (61.4)
Respondents’ source of information on small and sick newborn care during the COVID-19 pandemic						
Institution provided updated guidelines	81 (24.6)	25 (29.4)	65 (22.0)	6 (9.2)	7 (33.3)	184 (23.1)
Personally searched for information	224 (67.0)	52 (61.1)	158 (55.0)	15 (23.8)	14 (70.0)	463 (58.6)
Received information from colleagues or professional bodies	226 (67.6)	45 (52.9)	179 (62.3)	36 (57.1)	14 (70.0)	500 (63.3)
Received information from hospital or public health authorities	241 (72.1)	56 (65.8)	183 (63.7)	14 (22.2)	16 (84.2)	510 (64.6)
Respondents’ level of knowledge on care of newborns born to SARS-CoV-2 confirmed or suspected mothers						
Very clear	62 (18.6)	19 (22.1)	36 (12.0)	10 (15.4)	4 (19.0)	131 (16.3)
Mostly clear, but some areas of concern remain	111 (33.2)	38 (44.2)	126 (42.0)	34 (52.3)	7 (33.3)	316 (39.2)
Somewhat clear, but major issues remain	77 (23.1)	16 (18.6)	43 (14.3)	6 (9.2)	3 (14.3)	145 (18.0)
Some points clear but not confident	50 (15.0)	9 (10.5)	73 (24.3)	11 (16.9)	5 (23.8)	148 (18.4)
Not at all clear	34 (10.2)	4 (4.7)	22 (7.3)	4 (6.2)	2 (9.5)	66 (8.2)
Respondents’ work affected by COVID-19	292 (86.9)	74 (86.1)	237 (79.3)	48 (72.7)	19 (95.0)	670 (83.0)
Respondents' changes in practice due to COVID-19						
Reduced working hours	93 (27.6)	18 (20.6)	44 (14.7)	6 (9.0)	2 (9.5)	163 (20.1)
Always use PPE	263 (78.2)	63 (72.4)	205 (68.5)	50 (75.7)	18 (85.7)	599 (74.0)
Avoid practices that can increase transmission risk	271 (80.6)	54 (62.0)	161 (53.8)	14 (21.2)	11 (52.3)	511 (63.1)
No change in practice	16 (4.7)	3 (3.4)	37 (12.3)	1 (1.5)	0 (0)	57 (7.0)
Respondents’ fear for own health	314 (93.2)	78 (89.7)	231 (77.0)	58 (87.9)	15 (75.0)	696 (85.9)
Respondents’ higher stress level	305 (90.8)	80 (93.0)	256 (86.2)	58 (89.2)	19 (95.0)	718 (89.3)

*Differential number of missing values by variable.

†PPE is considered sufficient when items are available 100% of the time they are needed.

LatAm, Latin America; PPE, personal protective equipment; SEA, Southeast Asia.

The inadequate or erratic supply of personal protective equipment (PPE) was a frequently noted barrier; sanitiser and gloves were the most readily available, with 61.4% and 59.9% of respondents, respectively, reporting consistent access whenever these supplies were needed. There was a clear shortage of N95 masks and eye shields/protectors, with only 27.6% and 27.3% of respondents, respectively, reporting their availability at all times. Availability of PPE varied widely across regions, with Africa and SEA/Oceania being the most affected. Lack of PPE prevented providers from having close contact with mothers and their babies due to the risk of SARS-CoV-2 transmission. A paediatrician from Tanzania highlighted, ‘*the increased demand for PPE for nurses going to get expressed milk from quarantined mothers*’.

Lack of clarity regarding evidence-based maternal/newborn care guidelines was frequently noted by respondents, with only 16.3% reporting ‘very clear’ knowledge regarding care of neonates born to mothers with confirmed or suspected COVID-19. Sources of professional information on hospital care of small and sick newborns varied greatly and across regions (table 1). Institutional guidelines for small and sick newborn care and KMC were reported as available by 23.1% of respondents. Information on COVID-19 was mainly derived from internet searches (58.6%), colleagues and professional bodies (63.3%), and hospital or public health authorities (64.6%).

Knowledge gaps for the wider community and families regarding COVID-19 were also reported to affect access to care and duration of hospitalisation. Some respondents suggested that changes in behaviours emerged from fears caused by lack of knowledge or awareness regarding transmission risk and hygiene practices, with others noting that ‘*Families don't want to stay in hospital even after receiving counselling*’ (neonatal nurse, India). A community health worker from India reported, ‘*In the pandemic, baby’s family [may] refuse to go to the hospital; they say, ‘our child will [get] sick [due] to Corona*’’.

### Experiences and voices from neonatal care providers

Most respondents’ (83.0%) work has been affected by COVID-19, with 89.3% reporting higher than usual stress levels and 85.9% fearing for their own health. Across regions, 93.2% of African respondents reported fearing for their own health, while 93.0% of respondents in Latin America and the Caribbean reported higher than usual stress levels (table 1). Stress was primarily related to staff shortages due to infection, self-isolation or reallocation to COVID-19 wards, which resulted in an increased workload among the remaining neonatal care providers. A neonatologist from Ecuador reported that 40% of personnel working in his/her facility contracted SARS-CoV-2, ‘*affecting the number of personnel and the working hours*’. Most health providers reported apprehension related to COVID-19, including fear of contracting and spreading the virus and ‘*fear and panic for the unknown*’ (nurse, Nigeria).

### Disruptions for neonatal care provision and processes

Substantial disruptions in the use and delivery of care were reported (table 2), with families reluctant to access and stay in facilities. Reductions in hospital births and neonatal admissions of more than 25% were reported by 25% and 20% of respondents, respectively, with larger reductions occurring in Asian countries (35% and 27%). Changes to newborn care were noted to include reallocation of unit space (14.6%) and reassignment of staff from newborn care to COVID-19-related or other duties (18.9%). Oxygen supplies for newborn care were also reported to be compromised. In addition, requests for early discharge by families were widely reported by respondents, resulting in 43.8% of babies being discharged earlier than usual. A Kenyan paediatrician described an increase in ‘*anxiety of mothers due to worry of contracting COVID while in hospital*’, leading many families to request early discharge. A respondent from the Dominican Republic wrote, ‘*The KMC ward has been closed because it shared the space with the COVID area*’. A paediatrician from South Africa wrote, ‘*A COVID outbreak in mother lodger and KMC wards resulted in closure of wards, and delay and hesitancy in reopening*’. Limited space was noted as a barrier to maintaining social distancing measures in many settings and additionally affected the quality of neonatal care in facilities. A paediatrician from Kenya reported, ‘*[Babies are] sharing incubators, radiant warmers and phototherapy spaces*’.

**Table 2:** Effect of the COVID-19 pandemic on small and sick newborn care, KMC practice, facility visitation and follow-up care

	Total, n (%)
Neonatal inpatient care during COVID-19 pandemic (n=623)	
Newborn unit admission capacity reduced	229 (36.7)
Newborn unit/KMC areas are reallocated (for COVID-19 care or other care)	91 (14.6)
Newborn unit/KMC staff are reallocated (for COVID-19 care or other areas)	118 (18.9)
Babies are discharged earlier than usual	273 (43.8)
Women/families refuse to stay in facilities that are marked as COVID-19 treatment centres	197 (31.6)
KMC practice during COVID-19 pandemic (n=623)	
KMC ward admission capacity reduced	172 (27.6)
Health workers more hesitant to promote KMC	150 (24.0)
Women/families more hesitant to practise KMC	105 (16.8)
Counselling/support focus shifted from KMC to hand hygiene, masks and social distancing	237 (38)
KMC practised with improved hand and respiratory hygiene (ie, masks and tissues)	321 (51.5)
Changes to KMC practice* (n=528)	
KMC practice has stopped	37 (7.0)
KMC is practised as normal	292 (55.3)
KMC is practised, but the daily duration of skin-to-skin contact is reduced	140 (26.5)
KMC is initiated and babies are discharged home early	163 (30.8)
KMC is practised in another area	23 (4.4)
NSCU/NICU visitation during COVID-19 pandemic (n=593)	
Mother and family allowed as usual	68 (11.4)
Mothers are permitted except for those who are COVID-19-positive	304 (51.2)
Mother and family are not permitted	24 (4.0)
Family members (other than mother) are not permitted	266 (44.8)
Visiting hours are restricted	237 (39.9)
Follow-up care during COVID-19 pandemic (n=435)	
Reduced space for follow-up clinic	100 (22.9)
Less staff to conduct follow-up clinic	142 (32.6)
Fewer appointments for each newborn	211 (48.5)
Follow-up schedule has been changed	178 (40.9)
Women/families reluctant to attend follow-up due to fear of infection	319 (73.3)
Reduced attendance due to logistical reasons (eg, public transport disruptions)	247 (56.7)
Home visits disrupted	95 (21.8)
Telephone follow-up visits have been started.	160 (36.7)

*Changes to KMC practice were reported by 528 respondents due to embedded skip logic within the survey.

KMC, kangaroo mother care; NICU, neonatal intensive care unit; NSCU, neonatal special care unit.

At the hospital level, newborn follow-up care has been seriously affected by the pandemic (table 2), with 48.5% of respondents reporting fewer appointments per newborn and 32.6% reporting less staff to conduct follow-up clinics. Attendance has been interrupted by logistical challenges related to lockdown restrictions, such as decreased availability of public transport (56.7%) and financial constraints. In addition, 73.3% of respondents indicated that families were reluctant to attend follow-up appointments due to fear of COVID-19. Many respondents are conducting follow-up by telephone to mitigate the impact of these disruptions, resulting in suboptimal monitoring of infant growth stemming from lack of comprehensive examinations. Community health workers conducting neonatal home visits described being rejected by families. An Indian outreach worker wrote, ‘*[The] child’s family members refused me to come (in)to their home because they said ‘you [are] working out of your home and may be exposed to the pandemic*’’.

### Disruptions for KMC practice and respectful maternity care

Early discharge and fear of providers to come into close contact with mothers were major barriers to the practice of KMC. KMC was reported to be practised routinely by 85% of respondents before the pandemic, compared with 55% during the pandemic. Changes to KMC practice included reduced duration of skin-to-skin contact (26.5%), earlier discharge (30.8%) and full disruption of KMC services (7%). Respondents mentioned concerns for the continuation of KMC in the community following discharge, especially as counselling and assistance to mothers had been reduced due to competing activities among healthcare providers or social distancing measures. A paediatrician from Nicaragua explained, ‘*We limit [KMC] training to mothers only, fathers are being excluded by effects of the pandemic*’. Restrictions in visitation policies and family involvement in the provision of care for small and sick newborns impacted the normal practice of KMC, with family members not present to act as surrogates or provide support to mothers. Access and visiting hour limitations were widely reported. A paediatrician from Indonesia wrote, ‘*Mothers rarely come to the perinatal room for routine KMC, as regulation limits people to enter the perinatal room from outside hospital*’. Access to neonatal units was restricted, with 51.2% of respondents reporting that only mothers (except those positive for SARS-CoV-2) were permitted access and 11.4% reporting that families had usual access. In settings where visitations were still ongoing, many respondents expressed concern that family members were not following proper infection prevention and control (IPC) measures, such as hand hygiene, mask wearing and social distancing.^15^

KMC practice varied greatly by maternal SARS-CoV-2 status: positive, negative, suspected COVID-19 or unknown (table 3). Routine KMC practice (with or without a face mask) was reported by 79.2% of respondents for SARS-CoV-2-negative mothers and 32.4% for SARS-CoV-2-positive mothers. For SARS-CoV-2-positive mothers, almost 12% of respondents reported that they totally separated the baby from the mother and fed the baby formula milk. About 10% reported that they did the same for COVID-19 suspect mother–baby dyads. Breastfeeding disruptions were also highlighted, with practices including counselling, milk expression and human milk banking either stopped or continued at limited capacity during the pandemic.

**Table 3:** Care of small newborns during the COVID-19 pandemic, by maternal SARS-CoV-2 status

	Mother SARS-CoV-2-positive N=519 n (%)	Mother suspected with SARS-CoV-2 N=543 n (%)	Mother SARS-CoV-2 unknown N=672 n (%)	Mother SARS-CoV-2-negative N=664 n (%)
Routine KMC practice (with or without mask)	168 (32.4)	196 (36.1)	413 (61.5)	526 (79.2)
Mother and baby stay together, direct breast feeding but no prolonged skin-to-skin contact	93 (17.9)	112 (20.6)	151 (22.5)	89 (13.4)
Mother and baby separated, except during breast feeding	70 (13.5)	67 (12.3)	48 (7.1)	25 (3.8)
Mother and baby separated, expressed breastmilk feeding by uninfected caregiver	127 (24.5)	116 (21.4)	47 (7.0)	19 (2.9)
Mother and baby separated, formula feeding by uninfected caregiver (no breastmilk)	61 (11.8)	52 (9.6)	13 (1.9)	5 (0.8)

KMC, kangaroo mother care.

### Reported solutions and enablers

**Figure 2:**
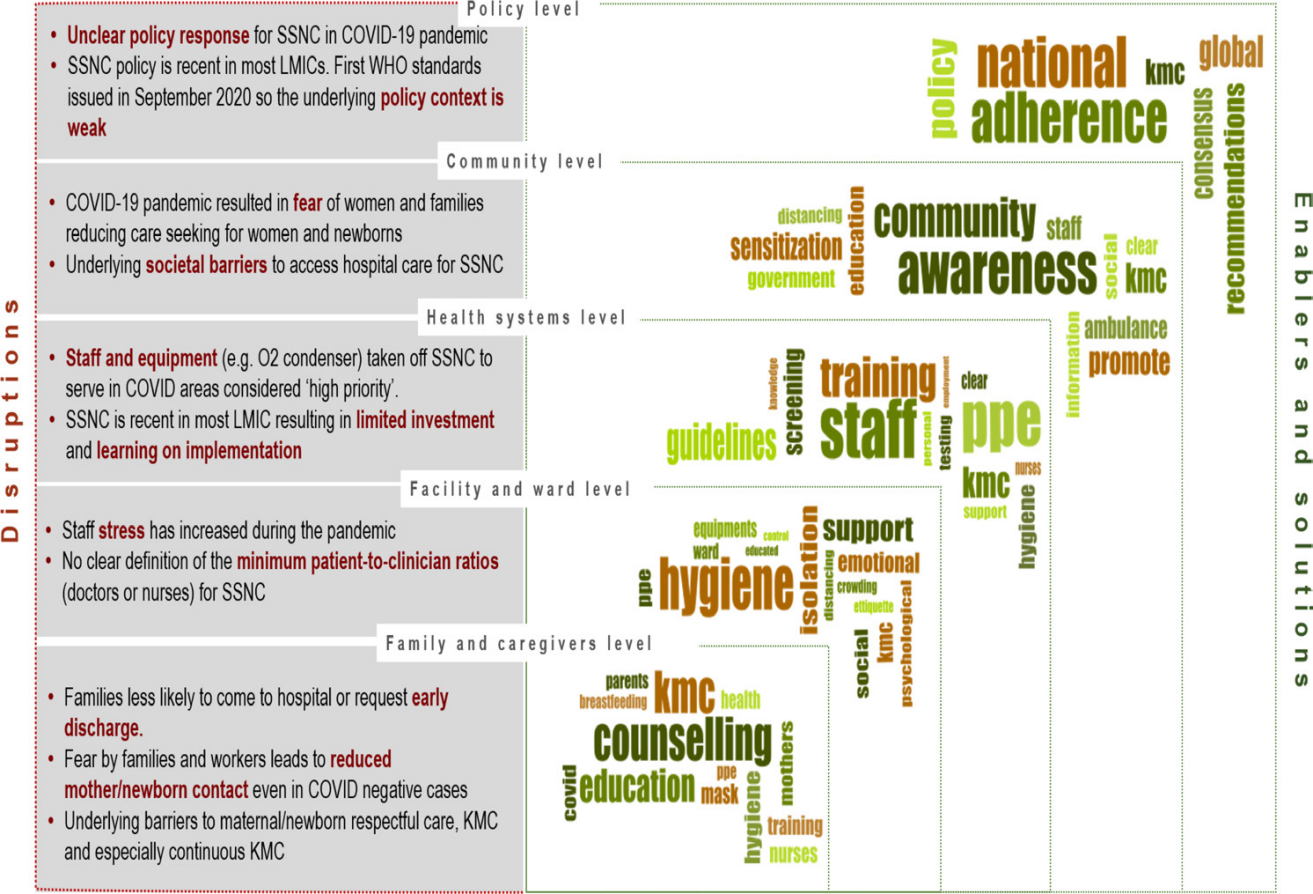
Synthesis of disruptions and underlying challenges, and analyses of solutions and enablers according to levels of care conceptual framework. LMIC, lowand middle-income country; KMC, kangaroo mother care; SSNC, small and sick newborn care.

Respondents suggested possible solutions to maintain service delivery for the small and sick newborn during the COVID-19 pandemic, with 55.4% of respondents reporting that they were currently implementing at least one of these solutions. We present their solutions by the levels of our conceptual framework: family and caregivers, facility and ward, health system, community and policy level (online supplemental appendix 3). A synthesis of disruptions/underlying challenges and reported solutions by these levels is given in figure 2.

At the family and caregivers’ level, most solutions aimed at promoting the safe continuation of lifesaving newborn interventions. A major theme was the need to increase awareness of caregivers with an emphasis on IPC, such as social distancing, correct mask wearing and hygiene practices. Many respondents stressed the importance of counselling sessions with mothers and families to educate them on adequate hygiene and safety protocols to increase their confidence to initiate and continue KMC and breastfeeding, even after discharge. Better education regarding follow-up care and reduction of transmission was also proposed to enable continued care and attendance at follow-up visits.

At the facility level, most solutions aimed at guaranteeing IPC measures, including intensifying the use of PPE, hand hygiene practice and increasing the number of cleaning staff. The allocation of isolation wards to separate Covid-19 suspected and positive babies and mothers was also proposed; a neonatologist from India suggested to create ‘*isolation wards for suspect or definite [COVID-19] cases separately. If space is a problem, then at least a barrier to separate between two cots*’. Avoiding overcrowding and respecting social distancing measures were mentioned frequently because of lack of space, and many recommended shifting to remote patient monitoring. A neonatal nurse from India suggested to ‘*install CCTV cameras in wards so that we [providers] can observe the patients even maintaining social distancing*’. Another solution that emerged was the need for increased education to healthcare workers with updated guidelines and clear protocols related to KMC and care of small and sick newborns, also suggesting that these trainings could serve as opportunities to provide emotional support to staff. A neonatologist wrote, ‘*daily meetings with frontline workers could provide an opportunity to address problems [faced during practice]*’.

At the level of health systems planning, many responses focused on increasing the number of personnel, reaching universal testing, ensuring supply provision and expanding infrastructure. In order to overcome health systems shortages such as limited personnel or reduction of space, respondents suggested innovative solutions like showing, ‘*videos demonstrating [practices] such as handwashing, breastfeeding and spoon feeding*’, or opening a ‘*neonatal COVID-19 query helpline*’. Many respondents encouraged the use of telemedicine and mobile health to maintain care coverage without overstretching available resources. A doctor recommended, ‘*Telephonic assistance or support should be provided after discharge from hospital as duration of stay is reduced so they may have not learnt proper KMC or breastfeeding technique*’.

Respondents underlined the imperative for a coordinated pandemic response including community interventions and greater government involvement. Many proposed the use of community sensitisation campaigns on COVID-19, using television or radio programmes to provide audiovisual education and tackle stigmatisation. Others recommended governmental policies to guarantee healthcare accessibility to vulnerable populations or to ensure provision of essential supplies, such as PPE. An administrator from the Philippines suggested that ‘*local government should provide transportation for mothers and newborns in order to increase access to health services*’. Moreover, a paediatrician from Tanzania highlighted the need to ‘*encourage local industry to manufacture affordable PPE*’.

## Discussion

While the direct mortality risk from SARS-CoV-2 infection in newborns is low, indirect effects of the COVID-19 pandemic are already increasing neonatal mortality in hospitals.^12^ Our survey provides sobering insights into disruption to care for small and sick newborns across the globe. We analysed responses from 1120 individuals in 62 countries from all world regions, encompassing all relevant cadres working in neonatal inpatient care as well as policymakers and public health professionals. The COVID-19 pandemic has caused serious disruptions at all levels of healthcare and particularly in LMICs, where the vast majority of newborn deaths occur. Our data suggest that coverage reductions for care at birth, and even more so for small and sick newborn care, continue at 20%–35%, although most of these countries were no longer in ‘*hard lockdowns*’.

These results underline the additional stressors of a pandemic overlaid on already overstretched health systems, especially in Africa and South Asia.^16^ Even 9 months into the pandemic, with the world facing a second wave, testing of pregnant women was reported to be unavailable by more than one in five African respondents. Even when testing was available, the lag between testing and results delayed or impacted safe clinical management. Gaps in PPE provision have been highlighted previously, including from high-income countries.^17^ Lack of availability of PPE was a major stressor for health personnel in our study.

The three-delay model^18^ is useful to explain how the pandemic results in disruptions,^19^ with delays in the decision to seek healthcare, delays in access and delays in receiving timely, high-quality care once at the facility. Delays in seeking or reaching care may be caused by fear of acquiring infection in hospitals and/or logistical effects of lockdown policies, such as curfews and transport restrictions.^16^ Pre-existing societal barriers also compound care-seeking for small and sick newborns and women with complications. Fatalism regarding newborns, especially those who are born preterm, is common in many cultures.^20–22^ In the context of the COVID -19 pandemic, even when women seek care, early discharge before full clinical stabilisation and preparedness is more common due to lack of family support and fear of SARS-CoV-2 infection. Improving community awareness about safety measures in health facilities, education and teleconsultation for follow-up care are some of the solutions that were suggested to reduce the occurrence and impact of these disruptions.

At the facility level, barriers to effective care include shortages of nurses and doctors whose function is further affected by an almost universal increase in stress, anxiety and fear, a recurring theme during previous outbreaks and this pandemic too. In the global maternity care survey, 90% reported increased stress.^14^ Increased stress stems from shortage of staff, overwork and lack of PPE. A recent survey of >2700 healthcare professionals from 60 countries found that half reported work-related burnout, as defined by a single measure of emotional exhaustion, and two-thirds indicated work impacting their quality of life and household activities during the pandemic.^17^ Availability of adequate PPE was associated with a 32% reduction in reported burnout among 314 respondents from LMICs.^17^ Provision of PPE and mental health support were recommended as potential solutions. Governments should perhaps consider prioritising provision of masks, eye shields and other PPE rather than items such as sanitisers, which can be replaced with soap and water hand washing.

Newborn care is a relative newcomer to global health,^23^ with programmatic focus only just beginning for hospital-based care of small and sick newborns.^9^ The WHO recently released standards for small and sick newborn care in the context of universal health coverage.^24^ Since this care is recent and considered low priority, with caregivers lacking power, in many facilities, the pandemic response has shifted essential equipment and workforce to other wards. Protecting and maintaining the staff, equipment and supplies in newborn units was strongly voiced among respondents in this survey.

Keeping mothers and their newborns together is fundamental for respectful and effective care. Our data show this principle is being disrupted for the COVID-positive mother and her newborn, and even for COVID-negative mother–baby dyads. KMC coverage is generally low, although increasing evidence shows that more rapid progress in scaling up is possible.^25^ The pandemic has further decreased KMC coverage, which could have a serious impact on survival of small newborns and could threaten global targets. Further, KMC continuity is adversely affected by restricted visitation polices, discharge before babies meet discharge criteria and limited post-discharge care. Strengthening counselling for KMC and breastfeeding and improving awareness of personal precautions could mitigate these effects to some extent. Unambiguous guidelines are urgently needed regarding small and sick newborn care in the context of the COVID-19 pandemic. Neonatal care, like care at birth, necessitates close interaction of health personnel with mothers and their babies, and trust, based on effective testing of both families and staff and provision of adequate PPE. A review examining COVID-19 guidelines on neonatal care for 17 countries highlighted variable quality and unsustainability of evidence,^26^ leading to uncertainties in policy and programmes.

Our results suggest that evidence-based care is being affected adversely, with two-thirds of providers reporting they would not allow mothers with confirmed or suspected SARS-CoV-2 infection to practise routine KMC, and nearly one-quarter reporting they would not allow breastfeeding. Health workers seemed unsure of guidelines for small and sick newborn care, even for breastfeeding, despite global educational campaigns by the WHO, UNICEF and others.^27 28^ Breastmilk is unlikely to be a route of SARS-CoV-2 transmission.^29–31^ Chambers and colleagues detected SARS-CoV-2 RNA in one of 64 milk samples from 18 infected women, with a negative viral culture for the positive sample, suggesting that RNA does not represent replication-competent virus.^29^ A case series reported that 4 of 12 neonates born to SARS-CoV-2-infected mothers tested positive within 48 hours of birth, after maternal symptom onset, of whom one was fed positive milk and subsequently tested negative despite exclusively breastfeeding while the mother was infected.^30^ A study among 185 neonates of infected mothers in Mumbai, India, reported that 12 (7%) tested positive for SARS-CoV-2 while bedding -in with their mothers, all of whom were healthy and thriving on exclusive breastmilk through 2 months of age.^32^ Available evidence suggests the benefits of breastfeeding on infant health, growth and development substantially outweigh the potential risk of SARS-CoV-2 transmission.

This study has several strengths. It is the first COVID-19 survey to focus on small and sick newborns—the most vulnerable users of any health system. It provides valuable insights into the specific disruptors of already tenuous care at different levels of the health system and potential solutions, which would help policymakers and administrators protect and sustain services. Learnings from this study would provide some guidance to make health systems more resilient to future pandemics. One limitation is that the online survey was only available in three languages, which could have affected the representativeness of respondents in some regions, as well as those working in extremely rural and remote areas where internet access is limited. However, we do have responses from 62 countries across all regions. Respondents from LMICs constitute the majority of responses, although we note that this may not be a major limitation considering LMICs have the greatest burden (98%) of neonatal deaths. During the 3 months the survey was open, countries were in different phases of the pandemic, and this may have affected responses. However, this could provide a diverse and comprehensive picture, which may be useful as the pandemic is still far from over and many countries are encountering varying waves of COVID-19. The survey was designed to highlight experiences of neonatal care providers, particularly those in hospitals; however, the voices of mothers, families and wider communities are also crucial. Our collaborative group is undertaking a multi-country qualitative study to better understand the perspectives of health workers, especially at the community level, as well as families of small and sick newborns.

## Conclusion

The COVID-19 pandemic has disrupted small and sick newborn care, including KMC, as well as caused high levels of stress among neonatal care providers. This paper sheds light on these effects and provides insights for policymakers. Management and allocation of newborn unit staff and essential equipment and supplies, including PPE, can and should be improved urgently. More attention must be placed on ensuring evidence-based practices, such as breastfeeding and KMC for all women and babies, including among SARS-CoV-2-positive mothers who are well enough. As a global health community, we need to act to protect the most vulnerable and prevent reversals of hard-earned gains in newborn survival, as well mitigate the wider impact on women, families and national development.
