# Supplementary material for: Small and sick newborn care during the COVID-19 pandemic: global survey and thematic analysis of healthcare providers’ voices and experiences
Source: BMJ Glob Health. 2021 Mar 14;6(3):e004347. doi: 10.1136/bmjgh-2020-004347 (PMC7959239; doi:10.1136/bmjgh-2020-004347)
Supplement: Supplementary data [file bmjgh-2020-004347supp001.pdf]

Appendix 1: Questionnaire in English

CARE OF THE SMALL BABIES AND KANGAROO MOTHER CARE IN THE COVID-19 PANDEMIC

Page 1: CONSENT

Thank you for taking this rapid survey to understand care of small babies and KMC during the COVID-19 pandemic. It will take less than 20 minutes to complete. Please go through the questions and mark all applicable choices.

1. Have you read the participant information sheet?
   a. Yes. I provide consent and voluntarily participate in the survey
   b. No

Page 2: INFORMATION SHEET

Please, read the information sheet before taking the survey. You can find it by clicking here

2. Have you read the participant information sheet?
   a. Yes. I provide consent and voluntarily participate in the survey
   b. No

Page 3: PERSONAL DETAILS/SETTING

3. What is your occupation?
   a. Nurse
   b. Neonatal nurse
   c. Paediatrician
   d. Neonatologist
   e. Medical doctor (no specialisation)
   f. Community health or outreach worker
   g. Administrator
   h. Policymaker
   i. Other (please specify)
4. Name your city/town
   ____
5. Name your country
   ____
6. What type of area do you work in?:
   a. Large city (>1 million people)
   b. Small city (100,000 to 1 million people)
   c. Town (<100,000 people)
   d. Village or rural area
   e. Refugee or emergency setting

f. Other- please specify ___________________________________

7. What level of hospital/organisation do you work in?:
    a. Tertiary
    b. Regional / general / secondary
    c. District
    d. Primary / sub district
    e. Not applicable
8. What type of hospital/organisation do you work in?
    a. Public or national health system
    b. Public academic (university or teaching)
    c. Private for profit
    d. Private not for profit eg Faith-based or mission
    e. Non-governmental
    f. Not applicable
    g. Other- please specify

Page 4: NEWBORN CARE CONTEXT

9. What is the highest level of newborn care provided at your facility?
    a. Neonatal intensive care (WHO level 3) - (Eg: ventilation)
    b. Newborn special care (WHO level 2) – (Eg: CPAP, IV fluids, assisted feeding)
    c. Essential newborn care – (Eg: thermal care, breastfeeding)
    d. Not applicable

10. What was the average number of **deliveries per month** that occurred in your facility prior to COVID-19 pandemic? (You could provide a range)

____

11. What was the average number of admissions to newborn unit per month in your facility prior to COVID-19 pandemic?

____

12. Do you provide Kangaroo Mother Care (KMC) in your facility?
    a. Yes
    b. No
    c. Not applicable

13. How many KMC beds do you have in your facility?

____

Page 5: COVID-19 PANDEMIC & EFFECTS

14. *How has the COVID-19 pandemic affected the number of* ***hospital births*** *in your setting?*
    a. *Increased*
    b. *Same-No change*
    c. *Reduced slightly (< 25%)*
    d. *Reduced significantly (25 – 50%)*
    e. *Reduced by more than half (> 50% reduction)*

15. *How has the COVID-19 pandemic affected the number of* ***newborn admissions*** *in your setting?*
    a. *Increased*
    b. *Same – no change*
    c. *Reduced slightly (< 25%)*
    d. *Reduced significantly (25 – 50%)*
    e. *Reduced by more than half (>50%)*

16. *Is your facility able to perform or order a COVID 19 test for pregnant women when they are admitted for delivery?*
    a. *Yes, routinely for all pregnant women admitted for delivery*
    b. *Yes, for pregnant women with specific risk factors*
    c. *Yes, for pregnant women with symptoms only / contact history*
    d. *Yes, for only elective CS*
    e. *No, we never test pregnant women admitted for delivery*

17. *Does your facility have a sign-posted area for screening of COVID-19 suspected cases?*
    a. *Yes*
    b. *No*

18. *Does your facility have isolation areas for confirmed / suspected cases of COVID-19?*
    a. *Yes*
    b. *No*

19. *How has COVID-19 affected staffing or team/shift composition in the newborn unit? (mark all that apply)*
    a. *No change*
    b. *Reduced staffing to supplement COVID units*
    c. *Additional support staff e.g cleaners*
    d. *Staff being absent due to symptoms (or quarantine)*
    e. *Change in shift hours*

20. *With regards to availability of PPE, during the last 10 times that the following PPE were needed, how often were they available?*
    a. *Gloves*
    b. *N95 Masks*
    c. *Eye protection (goggles, eye shield)*

d. *Aprons /gowns*
e. *Sanitizers*

21. *Did your institution / academic body provide updated guidelines for maternity care and/or small and sick newborn care during the COVID-19 pandemic?*
   a. *No*
   b. *Yes, updated guidelines for maternity care and routine well newborn care/breastfeeding only*
   c. *Yes updated guidelines for hospital care of small and sick newborns but not mentioning KMC*
   d. *Yes, updated guidelines for hospital care of small and sick newborns including KMC*

22. *Have you received information about COVID-19 from any other sources?(mark all that apply)*
   a. *No*
   b. *Personally searched for guidance and information*
   c. *Received information informally through colleagues/professional bodies*
   d. *Received information from hospital / public authorities*
   e. *Other- please specify*

   ____

23. *How would you describe your level of knowledge about providing care to neonates born to mothers with confirmed or suspected COVID-19?*
   a. *Not at all clear*
   b. *Some points clear but not confident in what I need to do*
   c. *Somewhat clear but major issues remain*
   d. *Mostly clear but some areas of concern remain*
   e. *Very clear*

24. *What do you think are the most urgent areas for health worker training/education for newborn care/KMC during the COVID-19 pandemic? (mark all that apply)*
   a. *Clarity and knowledge regarding safety of KMC and breastfeeding during COVID-19*
   b. *Counseling mother and family*
   c. *Proper use of PPE and measures of personal safety*
   d. *Management of COVID-19 positive mothers and their babies*
   e. *Other –Please specify*

   ____

25. *Has your work been affected by the COVID-19 pandemic?*
   a. *Yes*
   b. *No*

26. *How has the pandemic affected your personal level of stress?*
   a. *Same as usual*

b. *Somewhat higher than usual*
c. *Substantially higher than usual*

27. *Do you fear for your own health during the pandemic ?*
    a. *Yes*
    b. *No*
28. *How has the pandemic changed your practices?(mark all that apply)*
    a. *No change in practice*
    b. *Always use personal protective equipment*
    c. *Avoid practices that can increase the risk of transmission*
    d. *Reduced my work hours*
    e. *Other, please specify*

    ____

29. *What measures have you taken to protect yourself form COVID 19? (Mark all that apply)*
    a. *Use of PPE (eg: mask, gloves, gowns, eye shield)*
    b. *Measures of hand hygiene, cough etiquette*
    c. *Reduced work days / work from home*
    d. *Other- specify*

    ____

Page 6: KANGAROO MOTHER CARE

30. *Was Kangaroo Mother Care (KMC) routinely practiced in your health care setting before the pandemic?*
    a. *Yes*
    b. *No*

31. *Which newborns are started on KMC (i.e., what criteria are used to determine eligibility)?*
    a. *Babies ≤ 2000 g and stable*
    b. *Babies < 2500 g and stable*
    c. *Babies < 2000 g stable and unstable*
    d. *Babies < 2500 g stable and unstable*
    e. *Other, please specify*

    ____

32. *Where was KMC typically practiced before the pandemic? (mark all that apply)*
    a. *KMC ward*
    b. *Postnatal ward*
    c. *Newborn unit*
    d. *Other – specify*

    ____

33. *Is KMC still practiced now during the pandemic? (mark all that apply)*
    a. *KMC is not practised*
    b. *KMC is practised as normal*
    c. *KMC is practised, but the duration of KMC per day is reduced*
    d. *Babies are initiated in KMC but discharged home early*
    e. *KMC is practiced in another area (please specify)*

    ____

34. *How do you care for the small baby born to a* ***COVID 19 positive mother****?*
    a. *KMC as routine, (+ mask)*
    b. *Mother and baby stay together, breastfeeding per routine, but no prolonged skin-to-skin contact*
    c. *Mother and baby separated, except during breastfeeding*
    d. *Mother and baby separated, only expressed breast milk given*
    e. *Mother and baby separated, no breast milk given*
    f. *Not applicable*

35. *How do you care for the small baby born to a* ***COVID 19 suspect mother or has + contact history****?*
    a. *KMC as routine, (+ mask)*
    b. *Mother and baby stay together, breastfeeding per routine, but no prolonged skin-to-skin contact*
    c. *Mother and baby separated, except during breastfeeding*
    d. *Mother and baby separated, only expressed breast milk given*
    e. *Mother and baby separated, no breast milk given*
    f. *Not applicable*

36. *How do you care for the small baby born to a mother whose* ***COVID 19 status is unknown (not tested / all mothers) during the pandemic****?*
    a. *KMC as routine, (+ mask)*
    b. *Mother and baby stay together, breastfeeding per routine, but no prolonged skin-to-skin contact*
    c. *Mother and baby separated, except during breastfeeding*
    d. *Mother and baby separated, only expressed breast milk given*
    e. *Mother and baby separated, no breast milk given*

37. *How do you care for the small baby born to a* ***COVID 19 negative mother****?*
    a. *KMC as routine, (+ mask)*
    b. *Mother and baby stay together, breastfeeding per routine, but no prolonged skin-to-skin contact*
    c. *Mother and baby separated, except during breastfeeding*
    d. *Mother and baby separated, only expressed breast milk given*
    e. *Mother and baby separated, no breast milk given*

38. *Are the mother and family allowed to visit the small newborn in the special baby care unit/ NICU during the pandemic? (mark all that apply)*
    a. *Yes, as usual*
    b. *Visiting hours are restricted*
    c. *Mothers are permitted except for COVID -19 positive mothers*
    d. *Family members (other than mother) are not permitted*
    e. *Mother and family are not permitted*
    f. *Mother or family were not permitted even before the pandemic*

39. *How has the pandemic affected small newborn care, including KMC? (choose all that apply)*
    a. *Newborn unit admission capacity reduced*
    b. *KMC ward admission capacity reduced*
    c. *Newborn unit/KMC areas are reallocated (for COVID-19 care or other care)*
    d. *Newborn unit/KMC staff are reallocated (for COVID-19 care or other areas)*
    e. *Babies are discharged earlier than usual*
    f. *Health workers more hesitant to promote KMC*
    g. *Women/families more hesitant to practice KMC*
    h. *Women and families refuse to stay in facilities that are marked as COVID treatment centres*
    i. *Counselling and support focus shifted from KMC to hand hygiene, masks, social distancing*
    j. *Family members not able to be present for KMC support*
    k. *KMC practiced with improved hand hygiene and respiratory hygiene (i.e., masks, tissues)*
    l. *Other –please specify*

Page 7: SMALL BABY CARE AND FOLLOW UP

40. *Has the pandemic affected the following aspects of small and sick newborn care? (mark all that apply)*
    a. *Not affected*
    b. *Affected provision of oxygen*
    c. *Affected provision of CPAP*
    d. *Affected newborn feeding*
    e. *Affected monitoring including of blood sugar monitoring*
    Please specify the reasons why the care is affected (Eg: less oxygen/ concentrators available, CPAP device shortage, fewer nurses)

41. *Has the COVID-19 pandemic affected follow-up care for small and sick newborns?*
    a. *Yes*
    b. *No*

42. *How has the pandemic affected the follow up care?(mark all that apply)*
    a. *Reduced space for follow-up clinic*
    b. *Less staff to conduct follow-up clinic*
    c. *Fewer appointments for each newborn*

d. *Follow-up schedule has been changed*
e. *Women/families reluctant to come to hospital for follow-up due to fear of infection*
f. *Reduced attendance due to logistical reasons (e.g., public transport disruptions)*
g. *Home visits disrupted*
h. *Telephonic follow up visits have been started*
i. *Other - please specify*

Page 8: DISRUPTORS AND POTENTIAL SOLUTIONS

43. *As a health care provider, how do* ***you perceive*** *the KMC intervention during the pandemic? Would you encourage KMC during this pandemic?*
    a. *Overall, the risk is more than the benefit – should not promote KMC*
    b. *Overall, the risk is lower than the benefit – should promote KMC*
    c. *Risk is more than benefit only for COVID + mothers – withhold KMC only if mother is +*
    d. *Risk is lower than benefit even for COVID + mothers - continue KMC even if mother is +*

44. *List the key disruptors of small newborn care and KMC services in your facility/population that have been caused by the COVID-19 pandemic.*

Disruptor of small and sick newborn care/KMC

45. *List the potential solutions that could be adopted to address these key disruptors.*

Potential solution

46. *Is your facility/district implementing any solutions to overcome disruptions to small and sick newborn care and KMC services that have been caused by the pandemic?*
    a. *Yes*
    b. *No*

47. *Please elaborate on the solution your facility /district is implementing*

____

Page 9: CONTACT QUESTION

48. *Please select whether we may contact you via e-mail for any of the following purposes:*
    a. *If you would like to receive findings from this survey*
    b. *If you would like to be named in acknowledgement*
    c. *I would not like to be contacted*

49. *Please provide your e-mail address:*

____

Thank you for participating in the survey!
