# Supplementary material for: Small and sick newborn care during the COVID-19 pandemic: global survey and thematic analysis of healthcare providers’ voices and experiences
Source: BMJ Glob Health. 2021 Mar 14;6(3):e004347. doi: 10.1136/bmjgh-2020-004347 (PMC7959239; doi:10.1136/bmjgh-2020-004347)
Supplement: Supplementary data [file bmjgh-2020-004347supp002.pdf]

Appendix 2: Distribution of countries by region

AFRICA	LATAM AND CARIBBEAN	EUROPE AND NORTH AMERICA	ASIA EXCL. SEA	OCEANIA AND SEA
Swaziland	Antigua and Barbuda	Albania	Turkey	American Samoa
Guinea	Argentina	Andorra	Yemen	Australia
Ethiopia	Aruba	Armenia	Bahrain	Brunei Darussalam
United Republic of Tanzania	Bahamas	Austria	Iraq	Cambodia
Morocco	Barbados	Belgium	Jordan	Fiji
Democratic Republic of the Congo	Belize	Bosnia and Herzegovina	Kuwait	French Polynesia
Eritrea	Bolivia	Bulgaria	Lebanon	Indonesia
Angola	Brazil	Channel Islands	Oman	Kiribati
Côte d'Ivoire	British Virgin Islands	Croatia	Qatar	Lao PDR
Namibia	Cayman Islands	Cyprus	Saudi Arabia	Malaysia
Burkina Faso	Chile	Czech Republic	Palestine	Marshall Islands
South Africa	Colombia	Denmark	Syria	Micronesia, Fed. Sts.
Mauritania	Costa Rica	Estonia	United Arab Emirates	Myanmar
Sierra Leone	Cuba	Faroe Islands	Cyprus	Nauru
Gambia	Curacao	Finland	Israel	New Caledonia
São Tomé and Príncipe	Dominica	France	Azerbaijan	New Zealand
Egypt	Dominican Republic	Georgia	Armenia	Northern Mariana Islands
Kenya	Ecuador	Germany	Georgia	Palau
Djibouti	El Salvador	Gibraltar	Afghanistan	Papua New Guinea
Togo	Grenada	Greece	Bangladesh	Philippines
Senegal	Guatemala	Greenland	Maldives	Samoa
Nigeria	Guyana	Hungary	Pakistan	Singapore
Guinea-Bissau	Haiti	Iceland	Iran	Solomon Islands
Mali	Honduras	Ireland	Bhutan	Thailand
Mozambique	Jamaica	Isle of Man	Nepal	Timor-Leste
Botswana	Mexico	Italy	Sri Lanka	Tonga
Rwanda	Nicaragua	Kosovo	India	Tuvalu
Equatorial Guinea	Panama	Latvia	Democratic People's Republic of Korea	Vanuatu
Gabon	Paraguay	Liechtenstein	Mongolia	Vietnam
Tunisia	Peru	Lithuania	Republic of Korea	Cook Uskabds
Mauritius	Puerto Rico	Luxembourg	China	Niue
Madagascar	Sint Maarten	Moldova	Japan	
Uganda	St. Kitts and Nevis	Monaco	Kazakhstan	
Malawi	St. Lucia	Montenegro	Kyrgyzstan	
Benin	St. Martin	Netherlands	Tajikistan	

Liberia	St. Vincent and the Grenadines	North Macedonia	Turkmenistan	
Somalia	Suriname	Norway	Uzbekistan	
Cameroon	Trinidad and Tobago	Poland		
Niger	Turks and Caicos Islands	Portugal		
Central African Republic	Uruguay	Romania		
Sudan	Venezuela	Russian Federation		
Burundi	Virgin Islands	San Marino		
Zimbabwe		Serbia		
South Sudan		Slovak Republic		
Chad		Slovenia		
Congo		Spain		
Ghana		Sweden		
Cabo Verde		Switzerland		
Zambia		Ukraine		
Lesotho		United Kingdom		
Comoros		United States		
Algeria		Canada		
