# Supplementary figures and images for: Small and sick newborn care during the COVID-19 pandemic: global survey and thematic analysis of healthcare providers’ voices and experiences
Source: BMJ Glob Health. 2021 Mar 14;6(3):e004347. doi: 10.1136/bmjgh-2020-004347 (PMC7959239; doi:10.1136/bmjgh-2020-004347)

Appendix 3: Adapted socio-ecological framework

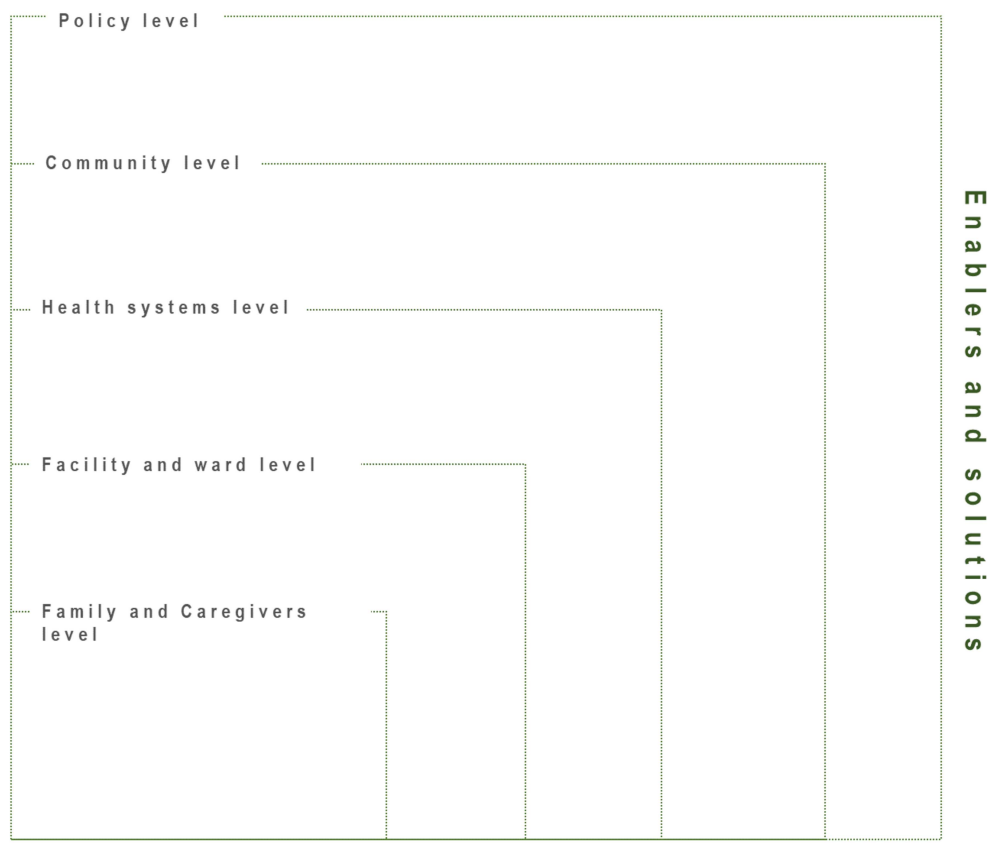

Supplement: Supplementary data [file bmjgh-2020-004347supp003.pdf]
